# Disruption of Axonal Transport in Motor Neuron Diseases

**DOI:** 10.3390/ijms13011225

**Published:** 2012-01-23

**Authors:** Kensuke Ikenaka, Masahisa Katsuno, Kaori Kawai, Shinsuke Ishigaki, Fumiaki Tanaka, Gen Sobue

**Affiliations:** 1 Department of Neurology, Nagoya University Graduate School of Medicine. 65 Tsurumai-cho, Showa-ku, Nagoya 466-8550, Japan; E-Mails: ikenakaman@med.nagoya-u.ac.jp (K.I.); kawaik@med.nagoya-u.ac.jp (K.K.); ftanaka@med.nagoya-u.ac.jp (F.T.); 2 Core Research for Evolutional Science and Technology (CREST), Japan Science and Technology Agency (JST), Saitama 332-0012, Japan; E-Mail: shinsuke.ishigaki@gmail.com (S.I.)

**Keywords:** axonal transport, dynactin-1, dynein, kinesin, neurofilament, motor neuron, amyotrophic lateral sclerosis, spinal and bulbar muscular atrophy, spinal muscular atrophy, hereditary spastic paraplegia

## Abstract

Motor neurons typically have very long axons, and fine-tuning axonal transport is crucial for their survival. The obstruction of axonal transport is gaining attention as a cause of neuronal dysfunction in a variety of neurodegenerative motor neuron diseases. Depletions in dynein and dynactin-1, motor molecules regulating axonal trafficking, disrupt axonal transport in flies, and mutations in their genes cause motor neuron degeneration in humans and rodents. Axonal transport defects are among the early molecular events leading to neurodegeneration in mouse models of amyotrophic lateral sclerosis (ALS). Gene expression profiles indicate that dynactin-1 mRNA is downregulated in degenerating spinal motor neurons of autopsied patients with sporadic ALS. Dynactin-1 mRNA is also reduced in the affected neurons of a mouse model of spinal and bulbar muscular atrophy, a motor neuron disease caused by triplet CAG repeat expansion in the gene encoding the androgen receptor. Pathogenic androgen receptor proteins also inhibit kinesin-1 microtubule-binding activity and disrupt anterograde axonal transport by activating c-Jun *N*-terminal kinase. Disruption of axonal transport also underlies the pathogenesis of spinal muscular atrophy and hereditary spastic paraplegias. These observations suggest that the impairment of axonal transport is a key event in the pathological processes of motor neuron degeneration and an important target of therapy development for motor neuron diseases.

## 1. Introduction

Motor neurons are highly specialized cells that possess the longest axons (more than 1 m long in human), which connect the soma with synaptic sites distant from the cell body. Moreover, although axons can represent >99% of the volume of a cell, protein and lipid syntheses occur almost exclusively in the cell body [1]. Therefore, active transport is required to supply the axon with newly synthesized materials and to transport neurotrophic factors and damaged organelles from the axon terminal to the cell body [2–4]. The major components of axonal transport are a group of specialized motor proteins and the cytoskeletal networks of microtubules and actin filaments. An increasing number of reports link defects in axonal transport with motor neuron degenerations, such as amyotrophic lateral sclerosis (ALS), spinal and bulbar muscular atrophy (SBMA), spinal muscular atrophy (SMA), and hereditary spastic paraplegias (HSPs) [1,5]. Moreover, several studies identified mutations in microtubule-based motor proteins of the kinesin and dynein superfamilies in certain hereditary forms of motor neuron diseases (MNDs): mutations in the genes encoding kinesin 1 motors (*KIF5A*) in certain types of HSPs [6]; two missense mutations in the dynein gene in the *Loa* and *Cra* mouse models of ALS [7]; and a mutation in the dynactin-1 gene linked to a familial form of lower motor neuron disease [8]. Dysfunctions of various membrane-associated proteins also cause motor neuron diseases by impairing the efficient transport of cargos such as mitochondria and endosomes. In this article, we summarize the current knowledge of the relationships between axonal transport defects and the pathogeneses of MNDs.

## 2. Motor Proteins and Motor Neuron Disease

### 2.1. Dynein/Dynactin Complex

Cytoplasmic dynein consists of two homodimerized heavy chains and multiple accessory proteins, including intermediate, light intermediate, and light chains [9], and is a major motor driving retrograde transport in cells. Dynein is involved in a variety of intracellular motile processes, including mitosis, maintenance of the Golgi apparatus, and the trafficking of membranous vesicles and other intracellular particles [5,10,11]. Two dynein mutant mouse strains, *Legs at odd angles* (*Loa*) and *Cramping1* (*Cra1*), were identified in a screen for genes involved in late onset MND [7]. Heterozygous mice carrying these mutations exhibit an age-related progressive loss of muscle tone and locomotor ability [7]. The mechanisms by which these mutations cause neurodegeneration are still discussed. Although Hafezparast *et al*. demonstrated that the *Loa* mutation causes axonal transport defect by using an assay for the visualization and quantitation of axonal retrograde transport of motor neuron cultures from *Loa/Loa* mouse based on a fluorescent fragment of tetanus toxin (TeNT HC), heterozygous knock out of dynein did not exacerbate, but ameliorated, axonal transport in a mutant superoxide dismutase 1 (SOD1) mouse model of ALS [7,12]. Moreover, Ori-McKenney *et al*. reported that neurodegeneration in the *Loa*^+/−^ mutant mouse is unlikely to result from dynein subunit dissociation or dissociation of dynein from its cargo, but from an altered interaction between dynein and microtubules [13]. On the other hand, another mutation of dynein, sprawling (*Swl*), induces an early-onset sensory neuropathy, but not progressive motor neuron loss, in the heterozygous knock out mice for this gene, indicating the perplexing relationship between dynein mutations and axonal transport [14]. In addition to the mutations in dynein, a G59S mutation in the P150^Glued^ subunit of dynactin (dynactin-1) has been identified in families with a slowly progressive, autosomal dominant form of lower motor neuron disease [8]. Dynactin is a multiprotein complex associated with dynein [15] and is required to attach dynein to its cargos [16]. Indeed, the dynactin depletion model of *Drosophila* exhibited disrupted axonal transport [17]. The G59S substitution occurs in the highly conserved CAP-Gly motif of dynactin-1, a domain that binds directly to microtubules [8] and slows transport of organelles *in vitro* [18]. The G59S-mutated protein also has an enhanced propensity to misfold and forms aggregates in which trapped organelles, such as mitochondria, are found. Although early degeneration of axons and neuromuscular junctions are the common features of three different mouse models of mutant G59S [19,20], whether an axonal transport defect is present in these models has yet to be elucidated. Lai *et al*. reported that the retrograde transport of neurofilaments and synaptophysin were affected in motor neurons of heterozygous knock-in mice with G59S mutant dynactin-1 [21]. However, Chevalier-Larsen *et al*. demonstrated the absence of axonal transport defects in their transgenic mouse model by performing a double ligation assay on sciatic nerves [19]. To resolve this discrepancy, further experiments, such as *in vivo* imaging of axons, are needed to analyze the transport of cargos, and it is also important to generate and analyze dynactin-1 depletion models.

### 2.2. Kinesin

The kinesin superfamily proteins (KIFs) comprise three major groups: *N*-terminal motor domain KIFs (*N*-KIFs), middle motor domain KIFs (M-KIFs), and *C*-terminal motor domain KIFs (C-KIFs), and are mainly responsible for the anterograde transport of the various materials to nerve terminals [22–24]. *KIF5A* encodes a neuron-specific, kinesin-1 heavy chain member of the kinesin superfamily, which has been implicated in MND. Mutations in the gene for the KIF5A subunit of kinesin 1 (formerly SPG10) are associated with an early-onset, pure form of HSP, exhibiting a dying-back neuropathy characterized by progressive weakness and spasticity of the legs [5,25]. The pathological mechanism underlying this mutant is the disruption of KIF5A’s ability to bind with microtubules, leading to a failure of adequate anterograde transport [26]. Because KIF5A motor proteins transport many cargos that are important for neurons, including neurofilaments, mitochondria, and SNARE proteins essential for synaptic vesicle docking [27,28], disruption of KIF5A motility results in dysregulation of axonal homeostasis. In addition to mutations in the kinesin family itself, modifications of the motor domain are also potential causes of MNDs [29].

## 3. Axonal Transport Defects and Cargo Accumulation

As a result of the decreased motility of motor proteins or their decreased binding to motor proteins, various cargos of motor proteins are accumulated in degenerated motor neurons. Among these we highlight three of the major cargos, neurofilaments, mitochondria, and autophagosomes, accumulations of which are the pathological hallmarks of various MNDs.

### 3.1. Neurofilaments

Neurofilaments are made up of three components, neurofilament light chain (NF-L), middle chain (NF-M), and heavy chain (NF-H). Neurofilament assembly in the axon is essential for establishing proper axon diameter, and its aggregates have long been recognized as a characteristic pathological change in MNDs [30–32]. Neurofilaments are transported along microtubules by kinesin and the dynein/dynactin-1 complex, and dysfunctions of these motor proteins lead to accumulation of neurofilaments in axons and somata, as observed in the *KIF5A* mutant mouse and *dynactin-1* mutant mouse [20,30,33,34]. Moreover, mutations in neurofilaments also cause motor neuron degeneration. Mersiyanova *et al*. reports that the A998C transversion in the first exon of *NF-L* results in Charcot-Marie-Tooth (CMT) type 2 [35]. The missense mutations in NF-L observed in CMT lead to disruption of axonal transport of neurofilaments and to the subsequent assembly and aggregation of NF-L in neuronal cells and in primary cultured neurons [36,37]. These studies strongly suggest that disruption of the axonal transport of neurofilaments is one of the underlying pathogenic mechanisms in the development of MNDs.

### 3.2. Mitochondria

Growing evidence suggests that mitochondrial damage is involved in motor neuron degeneration in CMT, HSP, and ALS. Local energy synthesis by mitochondria is essential to axonal homeostasis and its altered distribution and dysfunction play critical roles in axonal degeneration. Because mitochondria are transported by kinesin and the dynein/dynactin complex [24], defects in axonal transport result in abnormal accumulation of mitochondria. It has been shown that mitochondria accumulate in the axons of spinal motor neurons in mouse mutant SOD1 models [38–40] and in sporadic ALS (SALS) patients [41], suggesting an impairment of axonal transport in ALS. Indeed, in mutant SOD1 models, a defect in the axonal transport of mitochondria in sciatic nerves was directly observed during the presymptomatic stage [39]. Moreover, the presence of mitochondria with abnormal morphology, observed in both ALS models and SALS patients, indicates a poor recycling or degradation of abnormal mitochondria due to impaired retrograde transport [42–44].

It is important to assess whether the activation of mitochondrial mobility in axons has a beneficial effect on axonal degeneration in MNDs. It is proposed that increased mitochondrial transport might aid the efficient delivery of healthy mitochondria to axons and/or the removal of damaged mitochondria from the periphery. However, Zhu *et al*. reported that mutant SOD1 mice crossed with syntaphilin knock-out mice that have a higher proportion of axonal mitochondrial mobility, displayed no observable improvement in the deterioration of motor function or in disease progression and lifespan [45]. They suggested that mitochondrial dysfunction, rather than impaired mobility, may be more important for mutant SOD1 toxicity. On the other hand, a mouse model of mutant HSPB1-induced CMT exhibits impaired axonal transport of mitochondria, and treatment with histone deacetylase 6 inhibitors restored mitochondrial transport and attenuated the motor phenotype of this model [46].

### 3.3. Autophagosomes

The autophagy-lysosome pathway is responsible for the highly regulated recycling of intracellular contents, and it is well known that dysfunction of this pathway causes neurodegeneration [47,48]. Abnormal intraneuronal accumulation of autophagosomes is observed in various neurodegenerative diseases, including Alzheimer’s disease [49], Parkinson’s disease [50], and Huntington’s disease [51]. Because the autophagosome is a cargo that moves bidirectionally along microtubules powered by the kinesin family of motor proteins and the dynein/dynactin complex [52–55], the autophagosomes accumulates in those motor neurons with altered axonal transport. Human pathology and animal models of motor neuron disease also exhibited the same accumulations of autophagosomes, for example in SALS patients [56], the mutant dynein model (*Loa* mouse) [47], and in the mutant dynactin-1 mouse model [20]. Abnormal organelles, such as damaged mitochondria and dilated endoplasmic reticulum, are also observed in these motor neurons, and organelle accumulations are also observed in the neuritis of the Atg7^−/−^ mouse, which cannot form autophagosomes [48]. Therefore, disruption of autophagosomal transport resulting in the dysfunction of the autophagy system in axons is construed to be a causative mechanism of axonal degeneration.

## 4. MND and Axonal Transport Defects

MNDs affect various motor systems, including corticospinal upper motor neurons, lower motor neurons, neuromuscular junctions, and skeletal muscles. Impairments of these systems result in various motor phenotypes, such as muscle atrophy, weakness, and spasticity. Recent studies implicate axonal transport defects in the pathogeneses of MNDs (Table 1).

### ALS

Amyotrophic lateral sclerosis is a fatal neurodegenerative disease characterized by progressive loss of motor neurons [67]. Approximately 10% of ALS cases are familial (FALS) with inheritance patterns, while 90% are sporadic (SALS) with no known genetic defect [68,69]. There is at present no obvious consensus understanding of the pathogenic mechanism of SALS; however, the abnormal accumulation of neurofilaments, damaged mitochondria, and autophagosomes present in motor neurons of SALS patients [41,44,56,70], suggests that axonal transport defects might be implicated in the pathogenesis, but the molecular mechanisms causing axonal transport defects are still unclear.

**Figure 1:**
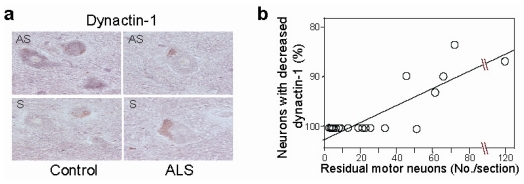
Dysregulation of *dynactin-1* in amyotrophic lateral sclerosis (ALS). (**a**) *In situ* hybridization of antisense (AS) and sense (S) *dynactin-1* probes in spinal motor neurons of control and ALS subjects; (**b**) Relationship between the number of residual motor neurons and the percentage of neurons with decreased *dynactin-1* (Reproduced from Jiang *et al*. [72]).

To uncover this mechanism, we created a motor neuron-specific gene expression profile in patients with SALS using microarray technology combined with laser-captured microdissection, the results of which were further verified by *in situ* hybridization and quantitative wide-ranging research activities [71]. Among the genes with dysregulated expressions, *dynactin-1* was markedly and widely downregulated in SALS motor neurons [71]. Furthermore, we found that the downregulation of *dynactin-1* occurs prior to the accumulation of phosphorylated-neurofilament, which is, again, a marker of defective axonal transport and is considered a rather early marker of neurodegeneration (Figure 1) [72]. The dramatic change in *dynactin-1* in patients with SALS seems to be specific for motor neurons, as *dynactin-1* expression was preserved in neurons in the dorsal nucleus of Clarke and the intermediolateral nucleus in the spinal cord, Purkinje cells of the cerebellum, and cortical neurons in the occipital cortex. By taking into account that mutations in *dynactin-1* cause MNDs, these results strongly suggest that the downregulation of *dynactin-1* in motor neurons may play a significant role in motor neuron degeneration in SALS.

### SBMA

**Figure 2:**
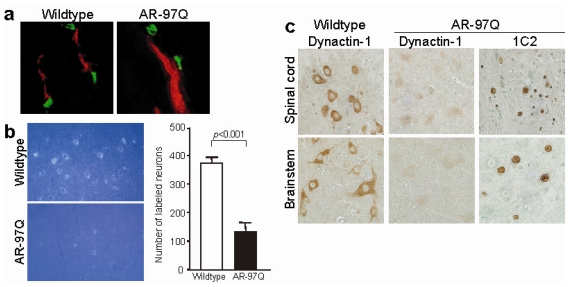
Disrupted retrograde axonal transport in a mouse model of spinal and bulbar muscular atrophy (SBMA). (**a**) Immunofluorescent anti-α-bungarotoxin (green) and anti-phospho NF-H (red) antibody staining of mouse skeletal muscle. Phosphorylated NF-H accumulates in the distal end of motor axons in the SBMA mice (AR-97Q); (**b**) Retrograde labeling of lumbar motor neurons by Fluoro-gold injection into the gastrocnemius muscle demonstrated decreased retrograde axonal transport in AR-97Q mice; (**c**) Immunohistochemistry shows decreased expression of dynactin-1 in motor neurons of spinal cord and brainstem of AR-97Q mice compared with wildtype mice. Accumulation of pathogenic AR is detected by 1C2, an anti-polyglutamine antibody (Reproduced from Katsuno *et al*. [62]).

Spinal and bulbar muscular atrophy, or Kennedy’s disease, is a hereditary neurodegenerative disease characterized by a loss of bulbar and spinal motor neurons [63,73]. The causative molecular defect in SBMA is the expansion of a trinucleotide CAG repeat, which encodes a polyglutamine tract in the first exon of the *AR* gene [74]. The resulting mutant AR forms aggregations in the nucleus and affects the expression of various genes by inhibiting transcription factors and coactivators [75,76]. Polyglutamine-expanded AR inhibits axonal transport via a pathway involving cJun *N*-terminal kinase (JNK) activation, phosphorylation of kinesin-1 heavy chain subunits by JNK, and inhibition of kinesin-1 function [29]. Interestingly, we found that *dynactin-1* is downregulated in the spinal motor neurons of SBMA patients and transgenic model mice (Figure 2) [62], which is also observed in SALS patients [71,72]. Fluoro-gold labeling revealed that retrograde axonal transport in SBMA mice was significantly impaired. Moreover, abnormal accumulations of neurofilaments and synaptic proteins were observed in the distal part of axons in these mice. The retrograde transport defects preceded the onset of motor symptoms, and were restored by hormonal interventions that prevent accumulations of mutant *AR* in the nucleus. The disruption of retrograde axonal transport was also documented in a knock-in mouse model of SBMA and in mice over-expressing wildtype *AR* in muscle [77], although this was not the case in another mouse model of SBMA [78].

### SMA

Spinal muscular atrophy is an autosomal recessive disease that selectively affects lower motor neurons and is classified into 3 forms: a severe form (type I; Werdnig–Hoffmann disease), an intermediate form (type II), and a juvenile form (type III; Kugelberg–Welander disease). SMA occurs in 1/6000 live births and is the leading genetic cause of infantile mortality in the USA. SMA is caused by the mutation or deletion of the “survival motor neuron 1” gene (*SMN1*), which plays an important role in RNA metabolism [79]. Reduced levels of SMN1 lead to pathological changes at neuromuscular junctions, including decreased synaptic vesicle density, synaptic transmission defects, and neurofilament accumulation [80]. In a mouse model of SMA, fast anterograde axonal transport is disrupted, providing the molecular basis for the synaptic pathology [81]. The levels of dynein in the sciatic nerve are also reduced in this mouse model, suggesting the concomitant involvement of retrograde axonal transport [81].

### HSP

The hereditary spastic paraplegias are a heterogeneous group of genetic disorders characterized by lower extremity spastic weakness due to retrograde degeneration of the corticospinal tracts and posterior columns, which is often accompanied by brisk reflexes, extensor plantar reflexes, and urinary urgency [82]. This disorder is classified into pure (uncomplicated) HSP and complicated HSP, depending on the presence of other neurological features in addition to spastic paraparesis. Spastic weakness is confined to the lower extremities in pure HSP, whereas complicated HSP is associated with mental retardation, ataxia, extrapyramidal signs, visual dysfunction or epilepsy. Among the causative proteins of HSPs, atlastin, spastin, and K1F5A regulate axonal transport [33,64–66]. While atlastin and spastin facilitate vesicle trafficking, KIF5A regulates anterograde axonal transport as described above.

## 5. Conclusions

The fine-tuning of axonal transport is crucial for the survival of motor neurons, while defects in axonal transport have been implicated in the pathogenesis of various disorders that affect motor systems. The elucidation of relationships between axonal transport and motor neuron degeneration is key to the development of molecular-targeted therapies and biomarkers for MNDs.

## Figures and Tables

**Table 1:** Gene mutations in motor neuron diseases (MNDs) affecting axonal transport.

MND type	Gene symbol	Protein	Protein function	Phenotype	Ref.
ALS1	*SOD1*	Cu/Zn superoxide dismutase	Detoxification enzyme	Varies among mutations from typical ALS type to atypical ALS	[57]
ALS2	*ALS2*	Alsin	Guanine nucleotide exchange factor (GEF) signaling; controlling endosomal dynamics	Juvenile onset, progressive muscle weakness and paralysis	[58]
ALS8 and SMA	*VAPB*	Synaptobrevin-associated membrane protein B (VAPB)	Vesicular trafficking; acts during ER-Golgi transport and secretion	Adult onset, slowly progressive upper and lower motor neuron disease. Phenotype varies from SMA type to ALS type	[59]
Lower motor neuron disease	*DCTN1*	Dynactin-1 (p150^glued^)	Retrograde axonal transport	Slowly progressive lower motor neuron disease	[8,18]
ALS	*CHMP2B*	Charged multivesicular body protein 2B (CHMP2B)	Vesicular trafficking; acts as a component of the ESCRTIII (endosomal secretory complex required for transport) complex	Lower dominant motor neuron disease	[60,61]
SBMA	*AR*	Androgen receptor	DNA-binding transcription factor	Slowly progressive lower motor neuron disease	[29,62,63]
SPG3	*ATL1*	Atlastin	Vesicular trafficking; a member of GTPase family, essential for axon formation and elongation	Early-onset pure, slow progression HSP	[64,65]
SPG4	*SPAST*	Spastin	An ATPase belonging to the AAA family acting for microtubule dynamics	Mainly pure HSP with variable onset	[66]
SPG10	*Kif5A*	Kinesin (K1F5A)	Anterograde axonal transport	Early onset progressive weakness and leg spasticity	[33]
